# Contamination-controlled cystectomy and ventricular wall reconstruction for an intramyocardial hydatid cyst of the left ventricle

**DOI:** 10.1016/j.xjtc.2026.102407

**Published:** 2026-05-02

**Authors:** Georg Daniel Duerr, Jan Beer, Robert Blumauer, Mikhail Khokhlunov, Tariq Abu-Tair, Christoph Kampmann

**Affiliations:** a Department of Cardiovascular Surgery, Section of Congenital Heart Surgery, University Medical Center, Johannes Gutenberg University, Mainz, Germany; b Department of Pediatrics, Pediatric Cardiology, University Medical Center, Johannes Gutenberg University, Mainz, Germany

**Figure:**
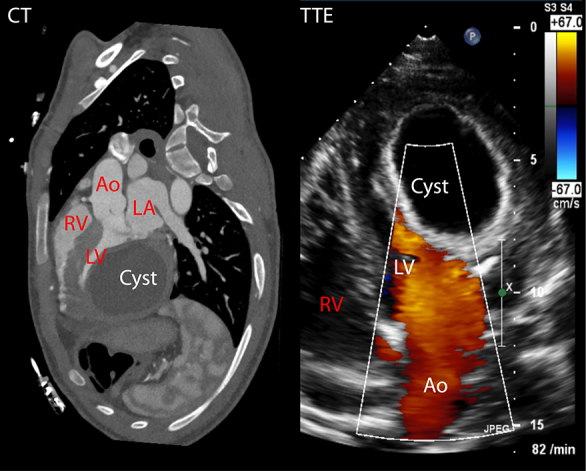
CT and TEE: intramyocardial LV cyst with thinned wall, narrow inflow/outflow.

**Central Message**

Contrast-enhanced CT (sagittal) shows a large intramyocardial LV hydatid cyst with a markedly thinned myocardial/endocardial layer and narrowed inflow/outflow. Apical 4-chamber TTE confirms the cyst.

Cardiac involvement in *Echinococcus multilocularis* cyst formation is rare (reported in approximately 0.02-2% of cases) but clinically important because of the risk of rupture, anaphylaxis, tamponade, and systemic embolization.^1^ The left ventricle (LV) represents one of the most frequent cardiac locations, consistent with coronary perfusion–related larval implantation.^2^ Diagnosis is primarily based on imaging.^2^^,^^E1^ Echocardiography is the first-line treatment imaging, whereas computed tomography (CT) can be used to define morphology and calcifications, and the use of cardiac magnetic resonance imaging (MRI) allows detailed tissue characterization and functional assessment. Serology may be negative and plays an adjunct role.^1^^,^^E1^ Because cardiac cysts can lead to catastrophic complications even when minimally symptomatic, surgical removal is generally recommended, with perioperative anthelmintic therapy frequently used to reduce recurrence risk.^1^^,^^3^, ^4^, ^5^ Operative strategies emphasize controlled decompression, scolicidal inactivation, and meticulous prevention of parasitic dissemination.^E2^ Technical descriptions of structured contamination-controlled cystectomy combined with ventricular reconstruction remain limited in the cardiac surgical literature. We therefore present a stepwise, contamination-controlled cystectomy of a large intramyocardial hydatid cyst of the LV that integrates ultrasound guidance, staged field isolation, and LV-wall reconstruction.

## Case Presentation

A 17-year-old male patient presented with several days of dizziness and intermittent palpitations. Initial evaluation for suspected myocarditis revealed a large cystic mass within the LV. Transthoracic echocardiography (Video 1) demonstrated a large cystic structure compressing the ventricular cavity without LV outflow tract obstruction and with preserved global systolic function. Cardiac CT showed a well-circumscribed intramyocardial fluid-density lesion in the anterolateral LV wall (∼5.6 × 8.0 × 5.3 cm) with focal calcification and significant compression of the ventricular lumen. Cardiac MRI confirmed a large intramyocardial cyst (∼7.7 × 5.5 × 5.7 cm) extending into the inferolateral myocardium with regional hypokinesia but preserved global function, eg, LV ejection fraction (∼57%). Screening for extracardiac disease showed no evidence of intracranial or liver echinococcosis on whole-body MRI, and findings on an ultrasound scan of the abdomen were unremarkable. Given the risks of rupture, anaphylaxis, and embolization, surgical removal was indicated.^1^ Preoperative anthelmintic therapy with albendazole was initiated according to published perioperative management strategies for cardiac echinococcosis.^3^ Albendazole therapy was continued postoperatively as part of a planned anthelminthic regimen aimed at reducing recurrence risk as described.^3^ The institutional review board waived the requirement for formal ethical approval. Written informed consent for surgery and video publication was obtained. Institutional ethical standards and patient confidentiality were maintained.

## Surgical Technique

After the induction of general anesthesia, a full median sternotomy was performed and the pericardium was opened. After systemic heparinization, cardiopulmonary bypass was established via ascending aortic and right atrial cannulation. A LV vent was placed through the right superior pulmonary vein. After aortic crossclamping, antegrade cold cardioplegia was administered until cardiac arrest. Epimyocardial ultrasound was used to localize the cyst and define safe access. To prevent contamination and parasitic dissemination, the operative field was widely isolated using pads soaked in 20% hypertonic saline and the exposed area was irrigated with hypertonic saline. Under brightness (B-) mode ultrasound guidance, the cyst was punctured and hydatid fluid was aspirated. The cavity was filled with hypertonic saline for scolicidal inactivation and left in situ for approximately 15 minutes. Immediately before we opened the cyst, additional hypertonic saline–soaked pads were positioned around the operative site as a secondary local contamination barrier. The cyst was then opened under continuous suction, and the germinative membrane of the cyst was carefully extracted in a controlled fashion. The cavity was inspected and debrided, with repeated hypertonic saline irrigation. No communication with the ventricular lumen or coronary vessels was identified. LV-wall reconstruction was performed using pledget-reinforced interrupted PROLENE mattress sutures with eversion of myocardial edges followed by continuous oversewing, again using PROLENE. Air evacuation was performed before final knot-tying, and the suture was sealed with BioGlue Surgical Adhesive (Artivion, Inc, USA). After deairing, the aortic clamp was removed and reperfusion initiated. The patient was weaned from cardiopulmonary bypass with transient inotropic support (Video 2).

## Results

The patient underwent fast-track anesthesia with extubation directly after skin closure in the operating room. Postoperative CT demonstrated total excision of the cyst with some air left in the cavity. Transthoracic echocardiography on postoperative day 12 (Video 3) showed slightly reduced biventricular function with regional dysfunction in the posterolateral wall, consistent with the LV suture but no shunt or pericardial effusion. Electrocardiogram demonstrated sinus rhythm without malignant arrhythmia. Early postoperative support included vasoactive therapy and remodeling therapy. Mobilization and recovery were uneventful. Histopathologic examination confirmed *E multilocularis* with periodic acid–Schiff stain–positive cyst membrane and regressive protoscolices. Microbiological analysis demonstrated numerous protoscolices in the cyst fluid without bacterial growth. The patient was discharged in stable condition with planned imaging follow-up.

## Discussion

Cardiac *E multilocularis* represents a very rare but potentially life-threatening manifestation of cystic echinococcosis.^1^ Multimodal imaging is central to diagnosis and operative planning, and the results of serologic testing may be negative.^2^^,^^E1^ Surgical treatment is recommended because of the risks of rupture, embolization, and progressive myocardial damage.^4^^,^^5^ Perioperative anthelminthic therapy is widely recommended in cardiac echinococcosis to reduce the risk of recurrence and secondary dissemination.^1^^,^^3^^,^^4^ In the present case, albendazole therapy was administered preoperatively and continued postoperatively as part of a structured treatment strategy, consistent with published perioperative management approaches in cardiac hydatid disease. Key operative priorities include contamination control, controlled decompression, scolicidal inactivation before cyst opening, and preservation of myocardial integrity.^E2^ Published surgical experiences emphasize that intraoperative rupture and dissemination represent the most critical risks and necessitate protective isolation and controlled evacuation techniques.^E2^ In the present case, a structured 2-stage contamination control strategy was applied: initial field isolation with hypertonic saline–soaked pads followed by a secondary local contamination barrier immediately before cyst opening. Most importantly, we used ultrasound-guided access, continuous suction during opening, and staged scolicidal inactivation combined with LV reconstruction to restore myocardial integrity and geometry.

The potential cytotoxic effects of hypertonic saline on myocardial tissue warrant consideration. Although intraoperative biopsy could theoretically assess such injury, it was not performed because of the high risk of ventricular wall perforation and biopsy-related myocardial or coronary injury in the setting of a thin residual myocardial layer.^E3^

Early postoperative ventricular dysfunction likely reflected myocardial stunning and remodeling after resection. Serial imaging confirmed the absence of residual parasitic disease. This case demonstrates a reproducible surgical strategy integrating contamination control, ultrasound-guided access, and ventricular reconstruction for intramyocardial hydatid cysts.

## Conclusions

Contamination-controlled cystectomy combined with structured ventricular reconstruction represents a safe surgical strategy for intramyocardial hydatid cysts and may reduce the risk of intraoperative dissemination and postoperative complications.

## Conflict of Interest Statement

The authors reported no conflicts of interest.

The *Journal* policy requires editors and reviewers to disclose conflicts of interest and to decline handling or reviewing manuscripts for which they may have a conflict of interest. The editors and reviewers of this article have no conflicts of interest.

## Supplementary Data

Video 1 Preoperative transthoracic echocardiography. Apical 4-chamber view demonstrating a large intramyocardial cystic lesion within the left ventricular wall causing compression of the ventricular cavity. The figure displays duplicate images: B-mode imaging on the left illustrating the cystic structure and its relation to the ventricular myocardium and color Doppler imaging on the right confirming the absence of intracystic blood flow and lack of communication with the ventricular cavity. Video available at: https://www.jtcvs.org/article/S2666-2507(26)00214-2/fulltext.

Video 2 Stepwise contamination-controlled cystectomy of an intramyocardial hydatid cyst of the left ventricle including epimyocardial ultrasound localization, staged hypertonic saline field isolation, ultrasound-guided aspiration, scolicidal inactivation, controlled cyst opening under continuous suction with membrane extraction, and ventricular wall reconstruction. Video available at: https://www.jtcvs.org/article/S2666-2507(26)00214-2/fulltext.

Video 3 Postoperative transthoracic echocardiography. Apical 4-chamber B-mode view obtained after surgical cystectomy and ventricular wall reconstruction. No residual cystic lesion is visible, and the reconstructed left ventricular wall is preserved without evidence of intracardiac shunt or pericardial effusion. Video available at: https://www.jtcvs.org/article/S2666-2507(26)00214-2/fulltext.
